# Monte Carlo calculated photon interaction coefficients for several body tissues

**DOI:** 10.1093/rpd/ncae006

**Published:** 2024-02-07

**Authors:** Aycan Sengul, Ahmet Bozkurt

**Affiliations:** Vocational School of Health Services, Medical Imaging Program, Akdeniz University, Antalya 07058, Turkey; Informatics Institute, Division of Computational Science and Engineering, Istanbul Technical University, Istanbul 34469, Turkey

## Abstract

Absorption of energy in body tissues because of radiation interactions may induce harmful outcomes such as cancer and hereditary effects due to a variety of damages in the integrity and activity of the cells. This study presents Monte Carlo calculated $\boldsymbol{\mu} /\boldsymbol{\rho}$, ${\boldsymbol{\mu}}_{\boldsymbol{en}}/\boldsymbol{\rho}$ and ${\boldsymbol{\mu}}_{\boldsymbol{tr}}/\boldsymbol{\rho}$ values of some common tissues and organs found in the human body (namely, adipose tissue, blood, bone-cortical, brain-grey/white matter, breast tissue, eye lens, lung tissue, muscle-skeletal, ovary, soft tissue and testes) as well as water for comparison purposes. The simulation model involves a monoenergetic point source producing a pencil beam where, depending on the parameter under study, particle flux, energy flux or absorbed dose from photon interactions are scored in the range of 10 keV to 20 MeV energy. The simulations were performed using the Monte Carlo package MCNP6.1 and provided $\boldsymbol{\mu} /\boldsymbol{\rho}$, ${\boldsymbol{\mu}}_{\boldsymbol{en}}/\boldsymbol{\rho}$ and ${\boldsymbol{\mu}}_{\boldsymbol{tr}}/\boldsymbol{\rho}$ values. The data produced in this study were compared with theoretical photon attenuation data from the XMUDAT database and demonstrated good agreement. The results, which are based on a simple model geometry and pure elemental compositions, indicate that this approach can be applied to evaluate $\boldsymbol{\mu} /\boldsymbol{\rho}$, ${\boldsymbol{\mu}}_{\boldsymbol{en}}/\boldsymbol{\rho}$ and ${\boldsymbol{\mu}}_{\boldsymbol{tr}}/\boldsymbol{\rho}$ in a broad energy range for any element, compound or mixture.

## Introduction

Radioisotopes that generate gamma or X-ray photons are utilised in a wide range of practical applications, from medical operations to beneficial uses in industrial and nuclear facilities. These indirectly ionising radiations cause ionisations and excitations in the material media they irradiate, transferring energy in the process. The significance of these interactions is determined by the material medium’s contents, and if the energy exchange is between the incoming photons and some medium of biological nature, the process of energy deposition may induce certain radiation effects. These effects can in turn be either deterministic or stochastic in nature and their severity is usually associated with the absorbed dose and the atomic composition of the biological material.

From a practical point of view, the harm that ionising radiation causes in human beings can be elaborated using some form of interaction coefficients^(1)^, such as the attenuation and absorption coefficients. These parameters are dependent on physical interactions and the energy of the incoming radiation and are usually expressed either microscopically in units of barn or more commonly in units of $c{m}^2/g$.

The mass attenuation coefficient ($\mu /\rho$) of an absorber provides an understanding of the probability of an incoming photon to undergo scatter or absorption interactions per unit distance and density of the material. It aids in estimating the type of interaction an ionising photon may go through whilst traversing an absorber. This, in turn, provides vital information for estimating the amount of energy to be deposited to the absorber. Hence, it is considered beneficial in obtaining a preliminary estimate of the material thickness to shield an ionising photon beam of known energy^(2)^. An overview of the research that reports mass attenuation coefficients for various materials over a wide range of photon energies can be found in^(3)^.

Although $\mu /\rho$ is useful for shielding estimates, it does not provide any direct information on radiation damage produced in irradiated materials as a result of radiation interactions. In biological absorbers, these effects may lead to certain damages in the integrity and activity of the cells in tissues^(4)^. The extent of these effects depends on such properties as the constituents of absorbing material and the amount of energy deposition both of which are better expressed by the energy transfer concept as defined in Attix^(5)^. This quantity is a function of radiant energy of uncharged particles entering and leaving the volume of interest and is expressed in terms of the kinetic energy of the liberated charged particles through definition of Kinetic Energy Released per Unit Mass (KERMA)^(6)^. For monoenergetic photons, KERMA is related to energy fluence through mass energy transfer coefficient (${\mu}_{tr}/\rho$) which is a characteristic of photon energy *E* and is defined as the energy per unit mass of material transferred to charged particles. It consists of the kinetic energy received by electrons from Coulomb interactions and spent through either collisions or radiative interactions. The first part is usually expressed as collision KERMA and is obtained from the net energy transferred to charged particles per unit mass.

For monoenergetic photons, collision KERMA is proportional to energy fluence where another quantity named as mass energy absorption coefficient (${\mu}_{en}/\rho$) can be seen as a constant of proportionality. ${\mu}_{en}/\rho$ is also a function of photon energy *E* and its calculation involves the energy transferred as kinetic energy to the charged particles that are created in the absorber as a result of photon interactions. Consequently, this parameter is largely preferred in dosimetric calculations and accounts for the mean energy of the incoming photons absorbed in the absorber. It provides itself as a significant tool for estimating absorbed dose since its calculation involves the energy transferred to the created charged particles as kinetic energy from photon interactions^(3)^.

There are reports in literature^(7–9)^ and an online database that provide $\mu /\rho$ and ${\mu}_{en}/\rho$ tables of photon interaction coefficients for various materials in a wide-ranging energies^(10)^. In addition, there is a computer code named XMUDAT^(11)^ that provides tabular evaluation of interaction coefficients for many elements, compounds or mixtures. For biological materials, however, there are some literature studies on evaluations of $\mu /\rho$, ${\mu}_{en}/\rho$ and ${\mu}_{tr}/\rho$ that are experimental evaluations based on detector measurements^(12–17)^. In addition, there are computational estimates^(^^18–25^^)^ based on either semi-analytical formulations or Monte Carlo calculations. The data produced, however, are typically for a restricted set of materials calculated at certain energy points. Especially for dosimetrically relevant body tissues, the data are scarce and are usually limited to tabular evaluations or computer codes such as XMUDAT^(11)^. For any material for which there is no direct data, the additivity rule may be used to extract individual $\mu /\rho$, ${\mu}_{en}/\rho$ and ${\mu}_{tr}/\rho$ values from the tables based on its elemental composition. This procedure, however, introduces some amount of error into calculations, as pointed out by Attix^(5)^. An alternative approach for obtaining photon interaction coefficients for any material in an energy range of interest will therefore serve as a vital tool in dosimetric and shielding studies.

The purpose of this paper is to apply the Monte Carlo technique to compute interaction coefficients for photons, and to provide a set of $\mu /\rho$, ${\mu}_{en}/\rho$ and ${\mu}_{tr}/\rho$ data for some body tissues (namely, adipose tissue, bone-cortical, brain-grey/white matter, breast tissue, lung tissue, muscle-skeletal, ovary, testes and soft tissue) as well as water for comparison. Based on a basic geometrical setting and pure elemental compositions, the Monte Carlo technique proposed in this study allows one to estimate photon interaction coefficients in a wide energy range for any element, compound or mixture.

## Materials and methods

 Monte Carlo method is a well-known statistical technique that is widely employed in a variety of scientific fields. It makes use of probability distributions and random numbers to calculate the average of a quantity for which a solution is either analytically or numerically difficult or unattainable. Because ionising radiation interactions with materials are probabilistic in nature and are typically characterised by microscopic cross-sections, Monte Carlo offers a convenient solution to radiation transport issues. Thus, a Monte Carlo code may be used in photon transport studies to compute dosimetric quantities such as flux, energy deposition, dose and so on, based on the source attributes and elemental compositions of the problem geometry^(26)^.

In this investigation, MCNP6.1 (Monte Carlo N-Particle version 6.1) was employed to simulate the geometry of the source, absorber and detector, as well as to estimate the interaction and detection of photons. The code is well recognised as a general-purpose radiation transport package developed at Los Alamos National Laboratory^(27)^ and is capable of transporting various types of source particles in three-dimensional geometries as well as handling a variety of detectors for scoring particle contributions. To handle particle interactions in a wide energy range and to derive desired particle properties, MCNP uses either physics packages or cross section libraries.

**Figure 1:**
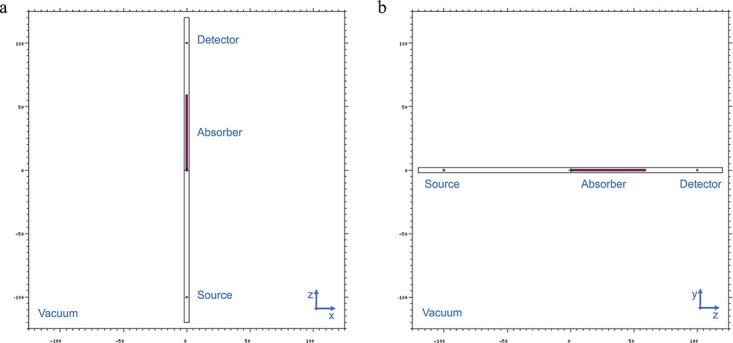
Views of the model geometry prepared using the MCNP plot function. (a) x-z (b) z-y.

A single input file was prepared to run MCNP simulations for the final computation of $\mu /\rho$, ${\mu}_{en}/\rho$ and ${\mu}_{tr}/\rho$ for the absorbers. A point photon source was positioned 100 cm from the origin of the coordinate system in a cylinder (*r* = 0.5 cm; *h* = 1 cm). It was used to generate monoenergetic and parallel photons to irradiate a cylindrical absorber (*r* = 0.5 cm; *h* = 1 mean free path) extended from the origin of the coordinate system towards the detector. The scoring volume was described as another small vacuum disc (*r* = 0.5 cm; *h* = 1 cm) which was positioned 200 cm away from the point source to serve as a detector to score the photons. As seen in Figure 1(a) and (b), an outer vacuum disc (*r* = 2 cm; *h* = 240 cm) served to encapsulate the cells in the problem geometry.

The cell flux feature of MCNP6.1 was utilised to record the number of particles passing through the detector volume and to derive the average of the total flux ($1/\left(c{m}^2\right)$) with ($I(x)$) and without (${I}_0$) each absorber in place. These values were then combined in Equation (1) to yield mass attenuation coefficients ($\mu /\rho$; in units of $c{m}^2/g$) for the samples and the photon energies studied.


(1) \begin{equation*} \frac{\mu }{\rho }=\left(-\frac{1}{x\rho}\right)\ln \left(\frac{I(x)}{I_0}\right) \end{equation*}

In this formulation, $x$ represents the absorber thickness and $\rho$ denotes the absorber’s density (in $g/c{m}^3$). The simulation setup modelled in this manner complies with the requirement of narrow-beam geometry and ensures no contribution from any scattered photons^(1)^.

The same geometrical setup was utilised for performing Monte Carlo simulations to compute ${\mu}_{en}/\rho$ and ${\mu}_{tr}/\rho$ values of the absorbers. However, in this situation, the volume of the absorber acted as the detector for scoring the dose and the flux. The simulations returned the energy flux (${\varPhi}_E$) and the absorbed dose ($D$) using *F4 (in MeV/cm^2^) and F6 (in MeV/g) tally features of MCNP, respectively. The ratio of absorbed dose and energy flux was then placed into corresponding equations (Equations (2) and (3)) to obtain the corresponding ${\mu}_{en}/\rho$ and ${\mu}_{tr}/\rho$ (in units of $c{m}^2/g$), respectively, for each sample at all investigated energies.


(2) \begin{equation*} \frac{\mu_{en}}{\rho }=\frac{D\ }{\varPhi_E} \end{equation*}

(3) \begin{equation*} \frac{\mu_{tr}}{\rho }=\frac{D^{\prime }\ }{\varPhi_E^{\prime }} \end{equation*}

In Equations (1) and (2), the simulation quantities were obtained using MCNP’s FT and FU cards with no collision, whilst in Equation (3) these features were not employed as indicated by the prime sign. In this manner, only the un-collided particles were considered to reach the detecting volume.

In this paper, 11 body tissues (namely, adipose tissue, blood, bone-cortical, brain-grey/white matter, breast tissue, eye lens, lung tissue, muscle-skeletal, ovary, soft tissue and testes) and water were investigated for estimating photon interaction coefficients. The density of each absorber is summarised in Table 1, along with the weight fractions of the elements H, C, N, O, Na, Mg, P, S, Cl, K, Ca and Fe that were based on the ICRU-44 compilation^(28)^ and ICRP-89 data^(29)^.

**Table 1:** Some properties of the body tissues investigated in this study.

**Sample**	**ρ (g/cm** ^**3**^ **)**	**H**	**C**	**N**	**O**	**Na**	**Mg**	**P**	**S**	**Cl**	**K**	**Ca**	**Fe**
Adipose Tissue	0.95	0.114	0.598	0.007	0.278	0.001			0.001	0.001			
Blood	1.06	0.102	0.11	0.033	0.745	0.001		0.001	0.002	0.003	0.002		0.001
Bone (Cortical)	1.92	0.034	0.155	0.042	0.435	0.001	0.002	0.103	0.003			0.225	
Brain (Grey/White Matter)	1.04	0.107	0.145	0.022	0.712	0.002		0.004	0.002	0.003	0.003		
Breast Tissue	1.02	0.106	0.332	0.03	0.527	0.001		0.001	0.002	0.001			
Eye lens	1.07	0.096	0.195	0.057	0.646	0.001		0.001	0.003	0.001			
Lung Tissue	1.05	0.103	0.105	0.031	0.749	0.002		0.002	0.003	0.003	0.002		
Muscle (Skeletal)	1.05	0.102	0.143	0.034	0.71	0.001		0.002	0.003	0.001	0.004		
Ovary	1.05	0.105	0.093	0.024	0.768	0.002		0.002	0.002	0.002	0.002		
Soft Tissue	1.06	0.102	0.143	0.034	0.708	0.002		0.003	0.003	0.002	0.003		
Testes	1.04	0.106	0.099	0.02	0.766	0.002		0.001	0.002	0.002	0.002		
Water	1	0.1119			0.8881								

The investigation comprised 28 photon energies ranging from 10 keV to 20 MeV for each sample, each representing a separate Monte Carlo run. The simulations, which involved no variance reduction technique, were performed for ${10}^6$ particle histories. On an Intel Xeon 2.1 GHz with 64 GB ram memory, each simulation took a few minutes to return a result with a statistical error of ˂0.1%.

## Results and discussion

In each simulation, MCNP6.1 was used to compute the average cell flux in a detector and the average energy flux and dose in the absorber volume. The tally results were then converted to $\mu /\rho$ using Equation (1), ${\mu}_{en}/\rho$ using Equation (2) and ${\mu}_{tr}/\rho$ using Equation (3). Table 2 shows Monte Carlo simulation results for total mass attenuation, energy absorption and energy transfer coefficients of 11 body tissues and water at 28 photon energies.

**Table 2:** Total mass attenuation, mass energy absorption and mass energy transfer coefficients (in cm^2^/g) determined from MCNP simulations for the samples.

**Energy**	**Adipose Tissue**			**Blood**			**Bone (Cortical)**			**Brain**		
**(MeV)**	$\boldsymbol{\mu} /\boldsymbol{\rho}$	${\boldsymbol{\mu}}_{\boldsymbol{en}}/\boldsymbol{\rho}$	${\boldsymbol{\mu}}_{\boldsymbol{tr}}/\boldsymbol{\rho}$	$\boldsymbol{\mu} /\boldsymbol{\rho}$	${\boldsymbol{\mu}}_{\boldsymbol{en}}/\boldsymbol{\rho}$	${\boldsymbol{\mu}}_{\boldsymbol{tr}}/\boldsymbol{\rho}$	$\boldsymbol{\mu} /\boldsymbol{\rho}$	${\boldsymbol{\mu}}_{\boldsymbol{en}}/\boldsymbol{\rho}$	${\boldsymbol{\mu}}_{\boldsymbol{tr}}/\boldsymbol{\rho}$	$\boldsymbol{\mu} /\boldsymbol{\rho}$	${\boldsymbol{\mu}}_{\boldsymbol{en}}/\boldsymbol{\rho}$	${\boldsymbol{\mu}}_{\boldsymbol{tr}}/\boldsymbol{\rho}$
0.01	3.258	2.933	2.94	5.499	5.093	5.129	28.42	26.81	27.68	5.391	5.013	5.034
0.015	1.079	0.8093	0.8149	1.739	1.438	1.453	9.006	8.39	8.614	1.705	1.41	1.42
0.02	0.5664	0.3249	0.329	0.8406	0.5829	0.5916	3.989	3.601	3.684	0.826	0.5703	0.577
0.03	0.3055	0.09492	0.09693	0.3841	0.1669	0.1708	1.327	1.07	1.095	0.3801	0.1629	0.1664
0.04	0.2389	0.04575	0.04679	0.2708	0.07445	0.07653	0.6637	0.4507	0.4636	0.2695	0.07277	0.07466
0.05	0.2116	0.03085	0.03142	0.2271	0.04478	0.04597	0.4231	0.2336	0.2415	0.2268	0.04395	0.04505
0.06	0.1966	0.02567	0.02599	0.2049	0.03334	0.03406	0.3139	0.14	0.1453	0.205	0.03289	0.03356
0.08	0.1794	0.02358	0.02365	0.182	0.02646	0.02673	0.2222	0.06903	0.07174	0.1824	0.02633	0.02658
0.1	0.1682	0.02433	0.02429	0.1689	0.02559	0.02567	0.185	0.04592	0.04745	0.1694	0.02558	0.02565
0.15	0.1493	0.02735	0.02723	0.1486	0.02747	0.02739	0.1474	0.03186	0.03234	0.1492	0.02756	0.02747
0.2	0.1363	0.02959	0.02946	0.1353	0.02945	0.02934	0.1304	0.03007	0.03025	0.1359	0.02956	0.02945
0.3	0.1181	0.03192	0.03182	0.1171	0.03164	0.03154	0.1109	0.03035	0.03037	0.1176	0.03177	0.03168
0.4	0.1058	0.03283	0.03276	0.1048	0.03251	0.03244	0.0987	0.03075	0.03074	0.1052	0.03265	0.03258
0.5	0.09657	0.03304	0.033	0.09561	0.03271	0.03267	0.08987	0.03079	0.03078	0.09602	0.03285	0.03281
0.6	0.08927	0.03291	0.03289	0.08837	0.03257	0.03255	0.08296	0.03059	0.03059	0.08876	0.03272	0.03269
0.8	0.07842	0.03218	0.03219	0.07762	0.03185	0.03185	0.07279	0.02984	0.02985	0.07795	0.03199	0.03199
1	0.07048	0.03115	0.03117	0.06977	0.03082	0.03085	0.06539	0.02886	0.02889	0.07007	0.03096	0.03098
1.25	0.06301	0.02977	0.02981	0.06237	0.02946	0.0295	0.05844	0.02758	0.02762	0.06264	0.02959	0.02963
1.5	0.05729	0.02845	0.0285	0.05672	0.02815	0.0282	0.05319	0.02637	0.02641	0.05697	0.02828	0.02833
2	0.04916	0.02619	0.02624	0.04871	0.02594	0.026	0.04585	0.02439	0.02443	0.04892	0.02605	0.02611
3	0.03932	0.02287	0.02292	0.03909	0.02275	0.0228	0.03723	0.0217	0.02174	0.03925	0.02284	0.02289
4	0.03358	0.02068	0.02072	0.03352	0.02067	0.02071	0.03239	0.0201	0.02013	0.03364	0.02075	0.02079
5	0.02978	0.01913	0.01917	0.02985	0.01924	0.01927	0.0293	0.01908	0.01909	0.02995	0.01929	0.01933
6	0.02708	0.018	0.01803	0.02727	0.0182	0.01822	0.02717	0.01841	0.0184	0.02734	0.01825	0.01827
8	0.02354	0.01649	0.0165	0.02391	0.01686	0.01686	0.02454	0.01767	0.01764	0.02396	0.01688	0.01689
10	0.02132	0.01555	0.01555	0.02184	0.01605	0.01604	0.02302	0.01734	0.01728	0.02187	0.01606	0.01605
15	0.01832	0.01433	0.01428	0.01909	0.01508	0.01501	0.0212	0.01723	0.01708	0.01908	0.01506	0.015
20	0.01688	0.01382	0.01374	0.01782	0.01474	0.01463	0.02057	0.0175	0.01725	0.0178	0.01471	0.0146
**Energy**	**Breast tissue**			**Eye lens**			**Lung tissue**			**Muscle (Skeletal)**		
**(MeV)**	$\boldsymbol{\mu} /\boldsymbol{\rho}$	${\boldsymbol{\mu}}_{\boldsymbol{en}}/\boldsymbol{\rho}$	${\boldsymbol{\mu}}_{\boldsymbol{tr}}/\boldsymbol{\rho}$	$\boldsymbol{\mu} /\boldsymbol{\rho}$	${\boldsymbol{\mu}}_{\boldsymbol{en}}/\boldsymbol{\rho}$	${\boldsymbol{\mu}}_{\boldsymbol{tr}}/\boldsymbol{\rho}$	$\boldsymbol{\mu} /\boldsymbol{\rho}$	${\boldsymbol{\mu}}_{\boldsymbol{en}}/\boldsymbol{\rho}$	${\boldsymbol{\mu}}_{\boldsymbol{tr}}/\boldsymbol{\rho}$	$\boldsymbol{\mu} /\boldsymbol{\rho}$	${\boldsymbol{\mu}}_{\boldsymbol{en}}/\boldsymbol{\rho}$	${\boldsymbol{\mu}}_{\boldsymbol{tr}}/\boldsymbol{\rho}$
0.01	4.281	3.934	3.942	4.812	4.455	4.464	5.44	5.063	5.081	5.338	4.96	4.982
0.015	1.374	1.093	1.099	1.527	1.24	1.247	1.716	1.421	1.43	1.688	1.394	1.404
0.02	0.6872	0.4392	0.4442	0.7491	0.4986	0.504	0.8295	0.5738	0.5803	0.8184	0.5636	0.5703
0.03	0.3394	0.126	0.1286	0.356	0.1421	0.145	0.3804	0.1635	0.1669	0.3772	0.1609	0.1644
0.04	0.2523	0.05793	0.05934	0.2583	0.06419	0.06578	0.2692	0.07288	0.07476	0.2678	0.07193	0.0738
0.05	0.2179	0.03667	0.03747	0.2202	0.03961	0.04053	0.2264	0.04394	0.04504	0.2256	0.0435	0.04458
0.06	0.1998	0.02882	0.02929	0.2005	0.03037	0.03092	0.2045	0.03285	0.03351	0.204	0.0326	0.03326
0.08	0.1801	0.02471	0.02486	0.1796	0.02519	0.02538	0.1819	0.02626	0.0265	0.1816	0.02615	0.0264
0.1	0.1682	0.02478	0.02479	0.1673	0.0249	0.02493	0.1689	0.0255	0.02556	0.1687	0.02544	0.0255
0.15	0.1487	0.02732	0.02722	0.1476	0.02717	0.02708	0.1487	0.02746	0.02738	0.1485	0.02743	0.02735
0.2	0.1356	0.02945	0.02933	0.1345	0.02923	0.02911	0.1354	0.02946	0.02935	0.1353	0.02943	0.02932
0.3	0.1174	0.03172	0.03162	0.1164	0.03145	0.03136	0.1172	0.03166	0.03157	0.1171	0.03163	0.03154
0.4	0.1051	0.03261	0.03254	0.1042	0.03233	0.03226	0.1049	0.03253	0.03247	0.1048	0.0325	0.03244
0.5	0.09592	0.03282	0.03278	0.09509	0.03253	0.03249	0.09569	0.03274	0.0327	0.0956	0.03271	0.03267
0.6	0.08867	0.03269	0.03266	0.0879	0.0324	0.03238	0.08845	0.0326	0.03258	0.08837	0.03257	0.03255
0.8	0.07788	0.03196	0.03197	0.07721	0.03168	0.03168	0.07769	0.03188	0.03188	0.07762	0.03185	0.03185
1	0.07001	0.03093	0.03096	0.0694	0.03066	0.03068	0.06983	0.03085	0.03088	0.06977	0.03082	0.03085
1.25	0.06259	0.02957	0.02961	0.06204	0.0293	0.02934	0.06242	0.02949	0.02953	0.06237	0.02946	0.0295
1.5	0.05691	0.02825	0.0283	0.05642	0.028	0.02805	0.05677	0.02818	0.02823	0.05672	0.02816	0.0282
2	0.04886	0.02602	0.02608	0.04845	0.0258	0.02585	0.04876	0.02597	0.02602	0.04871	0.02594	0.026
3	0.03914	0.02277	0.02282	0.03886	0.02261	0.02266	0.03913	0.02277	0.02282	0.03908	0.02274	0.02279
4	0.0335	0.02065	0.02069	0.0333	0.02053	0.02057	0.03355	0.02069	0.02073	0.03351	0.02066	0.0207
5	0.02978	0.01916	0.01919	0.02964	0.01909	0.01912	0.02988	0.01926	0.01929	0.02983	0.01922	0.01925
6	0.02714	0.01808	0.01811	0.02706	0.01805	0.01807	0.02729	0.01822	0.01824	0.02724	0.01818	0.0182
8	0.0237	0.01667	0.01667	0.02369	0.01669	0.0167	0.02393	0.01688	0.01688	0.02387	0.01683	0.01683
10	0.02157	0.0158	0.01579	0.02162	0.01588	0.01586	0.02186	0.01607	0.01605	0.0218	0.01602	0.016
15	0.0187	0.01471	0.01465	0.01885	0.01487	0.01481	0.0191	0.01509	0.01503	0.01903	0.01503	0.01496
20	0.01736	0.01429	0.01419	0.01757	0.01451	0.0144	0.01784	0.01476	0.01464	0.01776	0.01468	0.01457
**Energy**	**Ovaries**			**Soft tissue**			**Testes**			**Water**		
**(MeV)**	$\boldsymbol{\mu} /\boldsymbol{\rho}$	${\boldsymbol{\mu}}_{\boldsymbol{en}}/\boldsymbol{\rho}$	${\boldsymbol{\mu}}_{\boldsymbol{tr}}/\boldsymbol{\rho}$	$\boldsymbol{\mu} /\boldsymbol{\rho}$	${\boldsymbol{\mu}}_{\boldsymbol{en}}/\boldsymbol{\rho}$	${\boldsymbol{\mu}}_{\boldsymbol{tr}}/\boldsymbol{\rho}$	$\boldsymbol{\mu} /\boldsymbol{\rho}$	${\boldsymbol{\mu}}_{\boldsymbol{en}}/\boldsymbol{\rho}$	${\boldsymbol{\mu}}_{\boldsymbol{tr}}/\boldsymbol{\rho}$	$\boldsymbol{\mu} /\boldsymbol{\rho}$	${\boldsymbol{\mu}}_{\boldsymbol{en}}/\boldsymbol{\rho}$	${\boldsymbol{\mu}}_{\boldsymbol{tr}}/\boldsymbol{\rho}$
0.01	5.391	5.015	5.031	5.36	4.984	5.004	5.337	4.964	4.979	5.309	4.94	4.945
0.015	1.7	1.405	1.414	1.694	1.4	1.41	1.684	1.39	1.398	1.667	1.372	1.378
0.02	0.8226	0.5667	0.573	0.821	0.566	0.5727	0.8161	0.5603	0.5666	0.8076	0.5501	0.5556
0.03	0.3786	0.1613	0.1647	0.3779	0.1616	0.165	0.3768	0.1595	0.1629	0.3745	0.1556	0.1588
0.04	0.2686	0.07198	0.07383	0.2681	0.07217	0.07404	0.268	0.07127	0.0731	0.2676	0.0695	0.07125
0.05	0.2262	0.0435	0.04458	0.2257	0.04361	0.0447	0.226	0.04316	0.04422	0.2263	0.04224	0.04326
0.06	0.2045	0.03261	0.03326	0.204	0.03266	0.03332	0.2045	0.03243	0.03307	0.205	0.03193	0.03254
0.08	0.1821	0.02618	0.02642	0.1816	0.02618	0.02642	0.1822	0.02612	0.02636	0.183	0.02598	0.0262
0.1	0.1692	0.02548	0.02555	0.1687	0.02545	0.02551	0.1692	0.02547	0.02553	0.1701	0.02546	0.02551
0.15	0.1489	0.02749	0.02741	0.1485	0.02743	0.02735	0.149	0.02751	0.02742	0.1499	0.02762	0.02753
0.2	0.1357	0.02951	0.0294	0.1353	0.02943	0.02932	0.1358	0.02953	0.02942	0.1365	0.02968	0.02956
0.3	0.1174	0.03172	0.03162	0.1171	0.03163	0.03154	0.1175	0.03175	0.03165	0.1181	0.03192	0.03182
0.4	0.105	0.03259	0.03252	0.1048	0.0325	0.03243	0.1051	0.03262	0.03255	0.1057	0.0328	0.03273
0.5	0.09586	0.0328	0.03275	0.09561	0.03271	0.03266	0.09596	0.03283	0.03278	0.09649	0.03301	0.03297
0.6	0.08862	0.03266	0.03264	0.08837	0.03257	0.03255	0.0887	0.03269	0.03267	0.0892	0.03288	0.03285
0.8	0.07783	0.03194	0.03194	0.07762	0.03185	0.03185	0.0779	0.03197	0.03197	0.07834	0.03215	0.03215
1	0.06996	0.03091	0.03093	0.06976	0.03082	0.03085	0.07002	0.03094	0.03096	0.07042	0.03111	0.03114
1.25	0.06254	0.02954	0.02958	0.06237	0.02946	0.0295	0.06261	0.02957	0.02961	0.06295	0.02974	0.02978
1.5	0.05688	0.02823	0.02828	0.05672	0.02815	0.0282	0.05693	0.02826	0.02831	0.05725	0.02842	0.02847
2	0.04885	0.02601	0.02607	0.0487	0.02594	0.02599	0.04889	0.02604	0.02609	0.04917	0.02619	0.02624
3	0.0392	0.02281	0.02286	0.03908	0.02274	0.02279	0.03923	0.02283	0.02288	0.03946	0.02296	0.02301
4	0.03361	0.02073	0.02077	0.03351	0.02066	0.0207	0.03364	0.02074	0.02078	0.03384	0.02087	0.02092
5	0.02993	0.01929	0.01932	0.02984	0.01922	0.01925	0.02995	0.0193	0.01933	0.03014	0.01943	0.01946
6	0.02734	0.01825	0.01827	0.02724	0.01818	0.0182	0.02735	0.01826	0.01828	0.02754	0.01839	0.01841
8	0.02397	0.0169	0.0169	0.02388	0.01683	0.01683	0.02398	0.0169	0.01691	0.02415	0.01704	0.01704
10	0.02189	0.01609	0.01608	0.0218	0.01602	0.016	0.02189	0.01609	0.01607	0.02207	0.01623	0.01621
15	0.01913	0.01511	0.01504	0.01904	0.01503	0.01497	0.01912	0.0151	0.01504	0.0193	0.01525	0.01518
20	0.01786	0.01477	0.01466	0.01777	0.01469	0.01458	0.01786	0.01476	0.01465	0.01803	0.01492	0.0148

**Figure 2:**
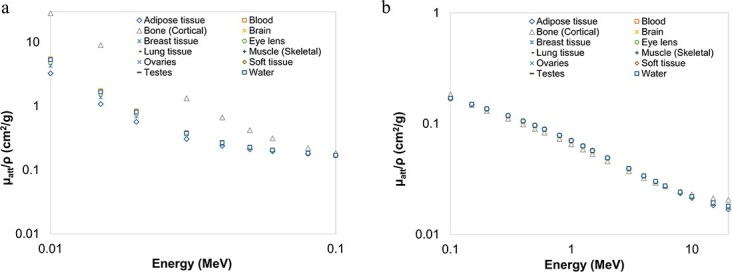
Mass attenuation coefficients (in cm^2^/g) of the body tissues as a function of photon energy computed with MCNP6. (a) 0.010-0.1 MeV (b) 0.10-20 MeV.

**Figure 3:**
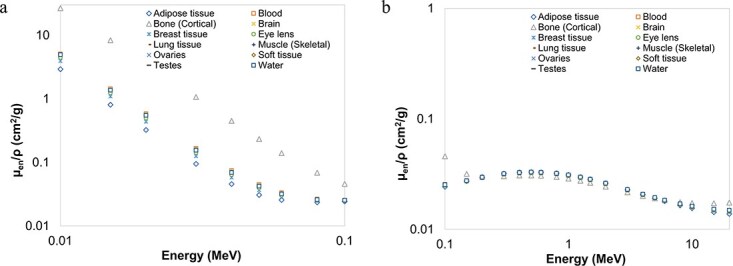
Mass energy absorption coefficients (in cm^2^/g) of the body tissues as a function of photon energy computed with MCNP6. (a) 0.010-0.1 MeV (b) 0.10-20 MeV.

**Figure 4:**
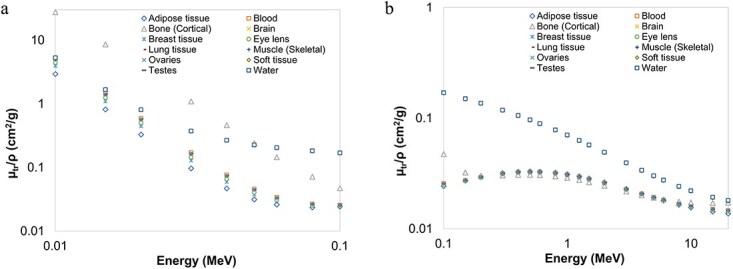
Mass energy transfer coefficients (in cm^2^/g) of the body tissues as a function of photon energy computed with MCNP6. (a) 0.010-0.1 MeV (b) 0.10-20 MeV.

Figures 2–4 provide graphical depictions of the obtained results for eleven body tissues and water as a function of energy. As seen in Figure 2(a), $\mu /\rho$ values always exhibit a sharp decrease up to roughly 50 keV. ${\mu}_{en}/\rho$ and ${\mu}_{tr}/\rho$ values of the investigated samples, on the other hand, display a comparable initial rapid reduction up to ~100 keV, as depicted in Figures 3(a) and 4(a), respectively. These behaviours may be explained by photoelectric absorption being the dominant interaction mode at these energies, particularly for elements of higher atomic number and thus such samples, in turn, possess higher interaction coefficients. For example, this behaviour is well demonstrated by cortical bone tissue, which contains some amount of phosphorus and calcium. After ~100 keV, however, the sudden decrease is replaced by roughly gradual fall for $\mu /\rho$ and a slower increase followed by a gradual decrease for ${\mu}_{en}/\rho$and ${\mu}_{tr}/\rho$, as demonstrated in Figures 2(b), 3(b) and 4(b), respectively. In this intermediate and higher energy range, all the samples follow an almost identical trend with little or no Z dependence since Compton scattering now becomes more significant. The incoming photons leave interaction sites with substantial amounts of energy and as a result the probability of energy absorption appears to increase with photon energy. This trend continues up to significantly higher energies of MeV level, after which the influence of atomic number becomes significant again.

When the results of MCNP6 simulations were compared with those of XMUDAT tables^(11)^ the discrepancies were determined to be minor for $\mu /\rho$ (˂0.5% in general) and ${\mu}_{en}/\rho$ (˂1% in general). The ${\mu}_{tr}/\rho$ simulations, on the other hand, returned results that agree with those of XMUDAT within ˂5%, which can be attributed to the characteristic peaks of the higher Z elements in the tissues. The corresponding R^2^ values of these correlations were thus equal to one, indicating a very acceptable agreement between the simulation findings and the corresponding XMUDAT data.

**Figure 5:**
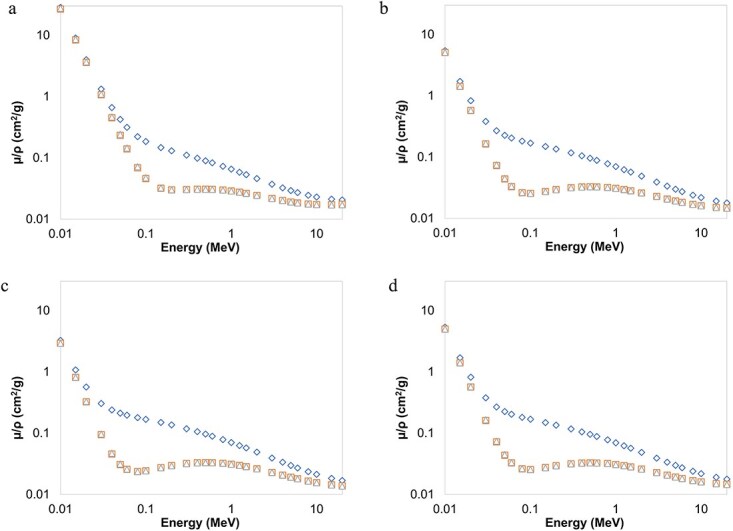
Interaction coefficients (cm^2^/g) of the samples as a function of photon energy computed with MCNP6 (◊: $\mu /\rho$;$\square$: ${\mu}_{en}/\rho;$ Δ: ${\mu}_{tr}/\rho$). (a) Bone (Cortical) (b) Lung tissue (c) Adipose tissue (d) Soft tissue.

Figure 5(a)–(d) detail the photon interaction coefficients from a radiation protection perspective and bring forth the difference values of $\mu /\rho$, ${\mu}_{en}/\rho$ and ${\mu}_{tr}/\rho$ for cortical bone, lung, adipose and soft tissues. At low energies where photoelectric absorption is more significant than Compton scattering and pair production interactions, these parameters have relatively closer values. Especially, ${\mu}_{en}/\rho$ and ${\mu}_{tr}/\rho$ results are similar within ~0.5% mostly, except at low energies where as much as 3% deviations are observed. With increasing photon energy, the values of the interaction coefficients begin to deviate, less remarkably for bone tissue compared to the other tissues because of its higher Z content. At the higher end of the spectrum, all tissues show a similar tendency with closer values of $\mu /\rho$, ${\mu}_{en}/\rho$ and ${\mu}_{tr}/\rho$. The distinctive deviation and relatively higher values of $\mu /\rho$ from those of ${\mu}_{en}/\rho$ and ${\mu}_{tr}/\rho$ for body tissues in the Compton region raise concerns about its usefulness as a radiation protection parameter. This is because only the amount of beam attenuation was included in its computation excluding the individual radiation effects that these photons may lead to. ${\mu}_{en}/\rho$ and ${\mu}_{tr}/\rho$, on the other hand, signify the mean energy of the incident photons absorbed by the absorber. Thus, they are regarded as better estimates of absorbed dose. For example, the calculation of ${\mu}_{en}/\rho$ involves the energy transferred to charged particles as kinetic energy via photon interactions. This may therefore be further related to the dose equivalent for biological formations by considering the linear energy transfer of the radiation environment.

## Conclusion

This study proposes a Monte Carlo approach for evaluations of photon interaction coefficients and provides the results of $\mu /\rho$, ${\mu}_{en}/\rho$ and ${\mu}_{tr}/\rho$ of some body tissues at various photon energies. The methodology follows computing either the attenuated photon flux at a distant detector for $\mu /\rho$, the absorbed dose and energy flux in an absorber for ${\mu}_{en}/\rho$ and ${\mu}_{tr}/\rho$. The simulations in the study provide results that are extremely close to the theoretical data of XMUDAT with minor discrepancies (˂0.5% in most cases). Therefore, the results indicate that Monte Carlo technique can be utilised as an alternative for calculating interaction parameters for any material for which data is not accessible in the literature. This approach can thus be utilised with confidence when measurements are difficult to perform, either because various photon energies are unavailable or because physical samples to be irradiated are difficult to manufacture at varying proportions.

## Data Availability

There is no data available to readers.
